# Patterns and Incidence of Pneumonitis and Initial Treatment Outcomes with Durvalumab Consolidation Therapy after Radical Chemoradiotherapy for Stage III Non-Small Cell Lung Cancer

**DOI:** 10.3390/cancers16061162

**Published:** 2024-03-15

**Authors:** Mizuki Sato, Kazumasa Odagiri, Yuya Tabuchi, Hiroaki Okamoto, Tsuneo Shimokawa, Yukiko Nakamura, Masaharu Hata

**Affiliations:** 1 Department of Radiation Oncology, Yokohama Municipal Citizen’s Hospital, Yokohama 221-0855, Japan; k7804140@yokohama-cu.ac.jp (K.O.);; 2 Department of Respiratory Medicine, Yokohama Municipal Citizen’s Hospital, Yokohama 221-0855, Japan; 3 Department of Radiation Oncology, Graduate School of Medicine, Yokohama City University, Yokohama 236-0004, Japan

**Keywords:** chemoradiotherapy, durvalumab, non-small cell lung cancer, immune checkpoint inhibitors, radiation pneumonitis, adverse effects

## Abstract

**Simple Summary:**

Definitive concurrent chemoradiation therapy (CCRT) is the standard of care for unresectable stage III non-small cell lung cancer (NSCLC), and durvalumab consolidation therapy after CCRT is now available following the results of the PACIFIC trial. However, its real-world clinical efficacy and impact on pneumonitis as a side effect have not been fully tested. In a retrospective analysis of 150 stage III NSCLC patients (durvalumab, n = 69; no-durvalumab, n = 81) who underwent CCRT at our institution, we found better progression-free survival and a higher incidence of pneumonitis grade ≥ 2 (G2) spreading beyond the irradiated fields in the durvalumab consolidation group than in the no-durvalumab group, but no change in ≥G3 severe pneumonitis. VS5 (lung volume spared from 5 Gy) was identified as a risk factor for pneumonitis ≥ G2 within the irradiated field in patients treated with durvalumab consolidation therapy. These results strongly encourage the use of durvalumab consolidation therapy in clinical practice.

**Abstract:**

Durvalumab consolidation after chemoradiotherapy for stage III non-small cell lung cancer (NSCLC) has become the standard of care. Single-center results were examined for treatment outcomes and patterns of pneumonitis in clinical practice. Patients with stage III NSCLC who underwent chemoradiotherapy at our institution (n = 150) were included. The patients were treated with chemoradiotherapy and durvalumab consolidation (Group D, n = 69) or chemoradiotherapy alone (Group N, n = 81). The overall survival (OS), progression-free survival (PFS), and the incidence of and risk factors for 12-month pneumonitis grade ≥ 2 (G2) were investigated. Two-year OS rates were 71.6% in Group D and 52.7% in Group N (*p* = 0.052). Two-year PFS rates were 43.0% in Group D and 26.5% in Group N (*p* = 0.010), although a propensity score matched analysis showed no significant difference. The incidence of 12-month pneumonitis ≥ G2 tended to be higher in Group D than in Group N (41.9% vs. 26.3%, *p* = 0.080). However, there was no difference in pneumonitis ≥ G3 rates (10.5% vs. 12.6%, *p* = 0.657). A multivariate analysis showed that the lung volume spared from 5 Gy (VS5) < 1800 cm^3^ was a risk factor for pneumonitis ≥ G2 in Group D. Durvalumab consolidation showed the potential to prolong PFS without increasing the severity of pneumonitis.

## 1. Introduction

Definitive concurrent chemoradiotherapy (CCRT) has long been the standard therapy for unresectable non-small cell lung cancer (NSCLC). However, the 5-year survival rate was only 10–30% [1,2]. Following the results of the PACIFIC trial, durvalumab consolidation therapy after CCRT was shown to improve outcomes [3,4,5] and has become the standard treatment for patients with unresectable stage III NSCLC who have undergone CCRT. In 2023, the results of the PACIFIC-R trial, which examined the real-world clinical efficacy of durvalumab consolidation, were reported, showing a favorable progression-free survival (PFS) [6].

Pneumonitis is a major side effect of radiotherapy for lung cancer and is known to occur in 20–30% of cases, with a predilection within 6 months after CCRT. Its prognosis is usually good; however, in rare cases, it may spread beyond the irradiated field or be life threatening. Durvalumab consolidation therapy may increase the incidence of pulmonary toxicity owing to various etiologies [7,8,9]. Before the durvalumab era, the percentage of lungs irradiated with 20 Gy (V20) and the mean lung dose (MLD) were associated with the occurrence of pneumonitis [10,11]. Some studies have reported that lower-dose areas, such as the percentage of lungs irradiated with 5 Gy (V5) or lung volume spared from 5 Gy (VS5), are especially important in patients receiving intensity-modulated radiotherapy (IMRT) [12,13,14], although V5 was not found to be a risk factor for pneumonitis in the secondary analysis of RTOG0617 [15]. An association between interstitial lung disease as the patient’s underlying disease and pneumonitis has also been noted [14,16,17]. However, existing pneumonitis prediction models have been reported to potentially underestimate the incidence of pneumonitis in patients who received durvalumab consolidation [18], so the actual risk factors in patients treated with durvalumab remain unclear.

Therefore, we investigated the clinical outcomes of patients treated with and without durvalumab at our institution, the pattern of pneumonitis as an adverse effect, and the risk factors of pneumonitis specific to the durvalumab group.

## 2. Materials and Methods

### 2.1. Study Design

Patients diagnosed with pathological stage III NSCLC who were treated with CCRT, with or without durvalumab consolidation therapy, at Yokohama Municipal Citizen’s Hospital between May 2013 and December 2022, were retrospectively analyzed. Consolidation therapy with durvalumab was initiated in July 2018. Patients with stages I–II and IV disease and those who received immune checkpoint inhibitors (ICIs) other than durvalumab as consolidation therapy were excluded. Data were collected using medical records and a dose-volume histogram (DVH) of the radiation treatment planning system. Regarding patient factors, data were collected on age, sex, Eastern Cooperative Oncology Group performance status (ECOG-PS), smoking history, and pulmonary fibrosis (PF) score (version modified by Tsujino et al. from the original) [14,19]. The PF score is outlined below. PF score 0, no fibrosis; PF score 1, interlobular septal thickening with no discrete honeycombing; PF score 2, honeycombing involving > 25% of the lobe; PF score 3, honeycombing involving 25–49% of the lobe. Data on clinical stage, histological type, presence of genetic mutations (including epidermal growth factor receptor [EGFR]), and programmed cell death ligand 1 (PD-L1) status were collected. Data on the prescribed dose, radiation technique (three-dimensional conformal radiotherapy [3D-CRT] or volumetric modulated arc therapy [VMAT]), treatment purpose (definitive treatment for primary disease or salvage treatment for postoperative recurrence), irradiation field (elective nodal irradiation [ENI] or involved field radiotherapy [IFRT]), and chemotherapy regimen were also gathered. As lung dose data, the V5 (%), V20 (%), MLD (Gy), VS5 (cm^3^), and total lung volume (TLV [cm^3^]) were collected for each case.

This study was approved by the Institutional Review Board of the Yokohama Municipal Citizen’s Hospital (No. 23-08-01) and was conducted in accordance with the Declaration of Helsinki.

### 2.2. Treatment

All patients were treated with 6- or 10-MV X-rays using a linear accelerator at 2 Gy once a day, five times a week, with concurrent platinum-based chemotherapy. Irradiation was performed after daily positional confirmation using cone-beam computed tomography (CT). Using 2- or 2.5-mm-thick treatment planning CT images, visible tumors and enlarged lymph nodes were identified for gross tumor volume (GTV). Positron emission tomography-CT was used as a standard for the identification of GTV. The clinical target volume (CTV) was defined as GTV plus 5 mm in all directions, and the prophylactic lymph node area was included in ENI cases. The internal target volume was created by adding a patient-specific margin of respiratory migration to CTV. Breath-holding irradiation was performed for patients with large respiratory migrations. The planning target volume (PTV) margin was ≥5 mm. For VMAT plans, the radiation treatment plans were designed so that 95% of the CTV volume would receive the prescribed dose and 95% of the PTV volume would receive 95% of the prescribed dose. All cases were irradiated with coplanar beams alone; non-coplanar beams were not used. All cases in which irradiation began after June 2020 were treated with VMAT, and all cases in which irradiation began after March 2020 were treated with IFRT. Previously, VMAT and IFRT were used in a limited number of cases. Patients who received durvalumab consolidation therapy were treated with a single 10-mg/kg intravenous infusion every 2 weeks for up to 12 months after the end of CCRT.

### 2.3. Endpoints

The overall survival (OS) and PFS were investigated as the treatment outcomes. The OS was defined as the time from the start of CCRT to the date of death. PFS was defined as the time from the start of CCRT to the date of death or recurrence. The incidence of pneumonitis, its risk factors, and its relationship with the lung dose were investigated. Treatment-related pneumonitis was defined as a clinically symptomatic pulmonary shadow occurring within 12 months of radiotherapy initiation, excluding those caused by obvious pulmonary infection. Pneumonitis within the irradiated field (in-field pneumonitis) was defined as cases in which the shadow was localized to the lung field through which the beam passed in 3D-CRT, or in the lung field in the rotational section of the arc in VMAT. Pneumonitis extending beyond areas of in-field pneumonitis was defined as out-of-field pneumonitis. In VMAT cases, since all VMAT cases in this study were irradiated with coplanar arcs alone, out-of-field pneumonitis was defined as pneumonitis in which the shadow cephalocaudally extends beyond the lung fields within the arc’s rotational range. The in-field and out-of-field data were retrospectively determined by two or more radiation oncologists. The grade of pneumonitis was determined by the attending respiratory physician at the time of its occurrence, according to the Common Terminology Criteria for Adverse Events version 5.0. Grade 1 pneumonitis was not investigated because of the difficulty in identifying its true time of occurrence.

### 2.4. Statistical Analyses

The baseline characteristics of the patients were compared using the Mann-Whitney *U* test for continuous variables and Fisher’s exact test for categorical variables. A propensity score matched (PSM) analysis was used to adjust for background factors. Kaplan-Meier curves were compared using the log-rank test in the univariate analysis for OS and PFS. For the cumulative incidence of 12-month pneumonitis, patients with no occurrence of grade ≥ 2 pneumonitis (pneumonitis ≥ G2) were censored at 12 months, and Kaplan-Meier curves were compared using the log-rank test. In the DVH analysis, lung doses were compared using the Mann-Whitney *U* test. Correlations between lung dose parameters were examined using Spearman’s rank correlation coefficient (ρ). The log-rank test and Cox proportional hazards regression models were used as risk factors for the development of 12-month pneumonitis, and factors with *p* < 0.05 in the univariate analysis were included in the multivariate Cox model. In addition, MLD and the PF score (an indicator of pre-existing interstitial lung disease) were included in the multivariate models as known risk factors. For MLD and VS5, receiver operating characteristic curves were created for each group, and cutoff values were determined with reference to the area under the curve and Youden’s index.

All tests were two-sided, and *p* < 0.05 was considered statistically significant. All statistical analyses were performed using EZR software, version 1.54, a graphical user interface for R [20].

## 3. Results

### 3.1. Patient Characteristics and Treatment

A total of 162 patients were diagnosed with NSCLC and treated with concurrent chemoradiotherapy (CCRT) at our institution. Six patients with stage I-II disease, one patient with stage IV disease, and five patients who received consolidation therapy with ICIs other than durvalumab were excluded, leaving 150 patients included in the analysis.

Of these 150 patients, 81 received CCRT alone (no durvalumab [Group N]) and 69 received CCRT followed by consolidation therapy with durvalumab (durvalumab [Group D]). Since July 2018, when the first case of durvalumab consolidation therapy was started, 17 patients did not receive durvalumab consolidation for various reasons and were thus included in Group N. The median follow-up was 19.2 (range: 3.3–53.7) months in Group D and 21.1 (range: 1.8–107.3) months in Group N. The median prescribed dose was 60 Gy in both groups, and all patients received a combination of platinum-based chemotherapies. In cases treated with ENI, the prophylactic area was irradiated with doses up to 40 Gy. Patient characteristics, including age, sex, ECOG-PS, smoking history, and PF score, did not markedly differ between the two groups. Tumor factors, including clinical stage, histological type, presence of genetic mutations, and definitive or salvage therapy, were also similar between the two groups. More patients in Group D were treated with VMAT (*p* < 0.001) and IFRT (*p* < 0.001) than those in Group N, and the PTV was significantly larger in Group N than in Group D (*p* < 0.001). However, there were no significant differences in the MLD, V5, V20, VS5, or TLV between the two groups. More patients in Group N did not have their PD-L1 levels measured than those in Group D (*p* < 0.001). The chemotherapy regimens differed between the two groups (*p* < 0.001). After PSM, 30 cases in each group were pair-matched. After PSM there were no significant differences in the patient characteristics. Details of the patients’ background characteristics and treatments are shown in Table 1.

In Group D, the median duration from the completion of radiotherapy to the start of durvalumab was 16 (range: 1–44) days, and the median number of doses was 12 (range: 1–27). At the time of analysis, 63 patients in Group D had ended durvalumab administration. Of these, 23 (36.5%) had completed the scheduled dose and 14 (22.2%) had completed the schedule without any interruptions. The reasons for the temporary interruption of durvalumab were pneumonitis in 19 patients, thyroid dysfunction in 1 patient, and other reasons in 3 patients. The reasons for the permanent discontinuation of durvalumab were tumor recurrence in 18 patients, pneumonitis in 13 patients, pulmonary infection in 2 patients, adrenal insufficiency in 1 patient, and other reasons in 5 patients.

### 3.2. The OS and PFS

**Figure 1:**
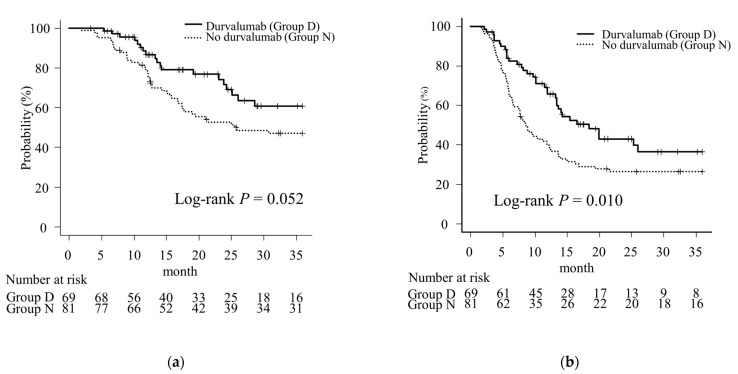
Kaplan-Meier curves of Stage III non-small cell carcinoma patients treated by chemoradiotherapy with or without durvalumab consolidation therapy. (**a**) The overall survival (OS). (**b**) The progression-free survival (PFS). Vertical lines on each line represent censored cases. Although there was no significant difference in the OS (**a**), the PFS was significantly better in the durvalumab group than in the no-durvalumab group (**b**).

During the observation period, 20 patients in Group D and 54 patients in Group N died, including 20 and 50 patients, respectively, who died of lung cancer. Disease recurrence was observed in 34 patients in Group D and 59 patients in Group N. The median OS was not reached but more than 36 months in Group D and 25.6 months in Group N. The median PFS was 18.4 months in Group D and 8.6 months in Group N. The 1- and 2-year OS rates were 86.6% and 71.6%, respectively, in Group D and 78.8% and 52.7%, respectively, in Group N (*p* = 0.052, Figure 1a). The 1- and 2-year PFS rates were 65.6% and 43.0% in Group D, respectively, and 40.4% and 26.5% in Group N, respectively; those in Group D were significantly better than those in Group N (*p* = 0.010, Figure 1b).

When 17 patients in Group N who started treatment after July 2018 (since the start of durvalumab use) were excluded, the median OS in Group N was 30.7 months, and 1-year and 2-year OS rates were 82.8% and 53.1%, respectively, which tended to be worse than in Group D, without significance (*p* = 0.127, Supplemental Figure S1a). The median PFS was 8.7 months, and the 1-year and 2-year PFS rates were 39.1% and 26.5%, respectively, which was significantly worse than in Group D (*p* = 0.013, Supplemental Figure S1b).

After PSM, the median OS was not reached (but >36 months) in Group D and 25.6 months in Group N. The median PFS was 15.4 months in Group D and 8.9 months in Group N. The 1- and 2-year OS rates were 89.9% and 68.8%, respectively, in Group D and 69.7% and 55.8% in Group N (*p* = 0.139, Figure S2a). The 1- and 2-year PFS rates after PSM were 73.0% and 38.3%, respectively, in Group D and 46.0% and 24.8% in Group N. There was a trend toward better PFS in Group D as was observed before PSM; however, the difference was not statistically significant (*p* = 0.091, Figure S2b).

### 3.3. Incidence of Pneumonitis

**Figure 2:**
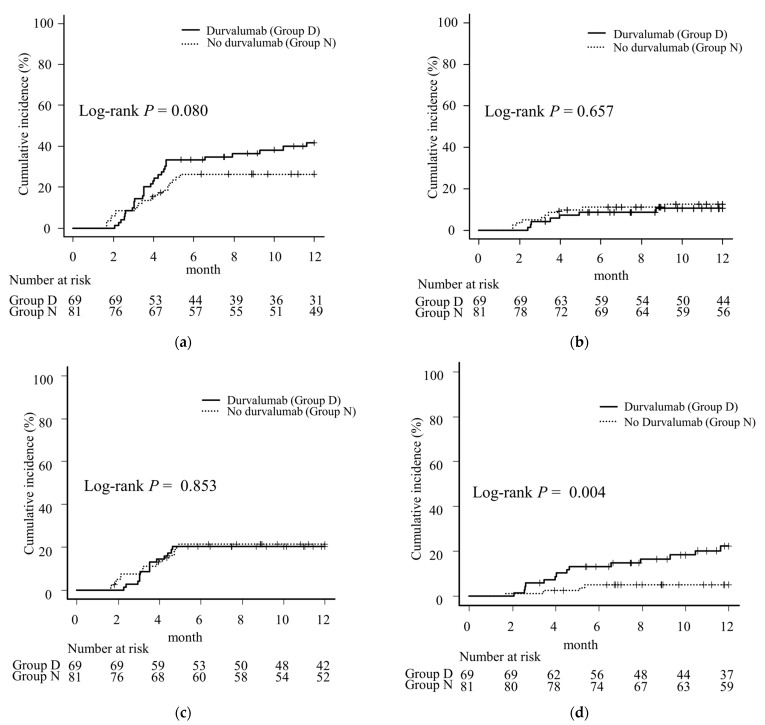
A comparison of the 12-month incidence of (**a**) pneumonitis grade ≥ 2 (≥G2), (**b**) pneumonitis grade ≥ 3 (≥G3), (**c**) pneumonitis within the irradiated field (in-field pneumonitis) ≥ G2, and (**d**) pneumonitis spreading beyond the irradiation field (out-of-field pneumonitis) ≥ G2 in Stage III non-small cell carcinoma patients treated with or without consolidation durvalumab after chemoradiotherapy. Vertical lines on each line represent censored cases. There was a trend toward more pneumonitis ≥ G2 in the durvalumab group than in the no-durvalumab group (**a**) but no marked difference between the groups in pneumonitis ≥ G3 (**b**). The incidence of in-field pneumonitis ≥ G2 was similar between the two groups (**c**). Out-of-field pneumonitis was more common in the durvalumab group than in the no-durvalumab group (**d**).

The number of patients who developed grade 2, 3, or 5 pneumonitis was 21, 7, and 0 in Group D, respectively, and 11, 9, and 1 in Group N, respectively. Of these, 14 cases in Group D and 4 cases in Group N were out-of-field pneumonitis. All cases of out-of-field pneumonitis were grade ≥3 (≥G3) in Group N, but only 4 of the 14 out-of-field cases were ≥G3 in Group D. The cumulative incidence of 12-month pneumonitis ≥ G2 tended to be higher in Group D than in Group N (41.9% in Group D, 26.3% in Group N, *p* = 0.080), but the difference was not significant (Figure 2a). However, the 12-month cumulative incidence of pneumonitis ≥ G3 did not differ between the groups (10.5% in Group D, 12.6% in Group N, *p* = 0.657) (Figure 2b). The difference in the cumulative incidence of 12-month in-field pneumonitis ≥ G2 between the two groups was similar (20.3% in Group D, 21.5% in Group N, *p* = 0.853) (Figure 2c), and out-of-field pneumonitis ≥ G2 was more common in Group D than in Group N (22.2% in Group D, 5.0% in Group N, *p* = 0.004) (Figure 2d). All cases of pneumonitis ≥ G2 occurred within six months in Group N, whereas five cases occurred after six months in Group D, all of which were out-of-field pneumonitis.

After PSM, the cumulative incidence of 12-month pneumonitis ≥ G2 tended to be higher in Group D than in Group N (36.7% in Group D, 23.9% in Group N, *p* = 0.394), but the difference was not significant (Figure S3a). The 12-month cumulative incidence of pneumonitis ≥ G3 still did not differ between the groups (13.3% in Group D, 10.4% in Group N, *p* = 0.727) (Figure S3b). The difference in the cumulative incidence of 12-month in-field pneumonitis ≥ G2 between the two groups was not significant (10.0% in Group D, 17.3% in Group N, *p* = 0.347) (Figure S3c). Out-of-field pneumonitis ≥ G2 tended to be more common in Group D than in Group N, as was observed before PSM; however, the difference was not statistically significant (27.0% in Group D, 7.1% in Group N, *p* = 0.056) (Figure S3d).

The MLD, lung V5, V20, VS5, and TLV in patients with pneumonitis ≥ G2 were compared between cases of out-of-field and in-field pneumonitis. While there were no significant differences in any parameters in Group N, patients with out-of-field pneumonitis tended to have lower MLD, V5, and V20 values than in comparison to patients with in-field pneumonitis in Group D, with significant differences in MLD and V20 (Table 2).

### 3.4. Univariate and Multivariate Analyses for Pneumonitis ≥ G2

MLD and VS5 were adopted as candidate risk factors for pneumonitis ≥ G2. V20 was not employed because of its strong positive correlation with MLD (ρ = 0.97). Since V5 had a relatively high correlation with MLD and V20 (ρ = 0.79 for MLD and ρ = 0.72 for V20); however, the correlation with VS5 was not so high (ρ = −0.60 for MLD and ρ = −0.56 for V20). VS5 was employed instead of V5 as an indicator for the low-dose range. To determine the cutoff value, ROC curves were created for VS5 and the occurrence of pneumonitis ≥ G2. The AUC was 0.62 and Youden’s index was 1810 cm^3^ for Group D (Figure S4a), while the AUC was 0.62 and Youden’s index was 1859 cm^3^ for Group N (Figure S4b). Therefore, VS5 = 1800 cm^3^ was adopted as the cutoff value. Using the same method, ROC curves were generated for MLD, the AUC was 0.52 and Youden’s index was 17.2 Gy for Group D (Figure S4c), while the AUC was 0.67 and Youden’s index was 14.1 Gy for Group N (Figure S4d), respectively. Since the AUC of the ROC curve for Group D was almost 0.5 and Youden’s index was also far from the median MLD for Group D, 17.2 Gy was considered inappropriate as a cutoff value. Therefore, MLD = 14 Gy was adopted as the cutoff value for both groups.

**Figure 3:**
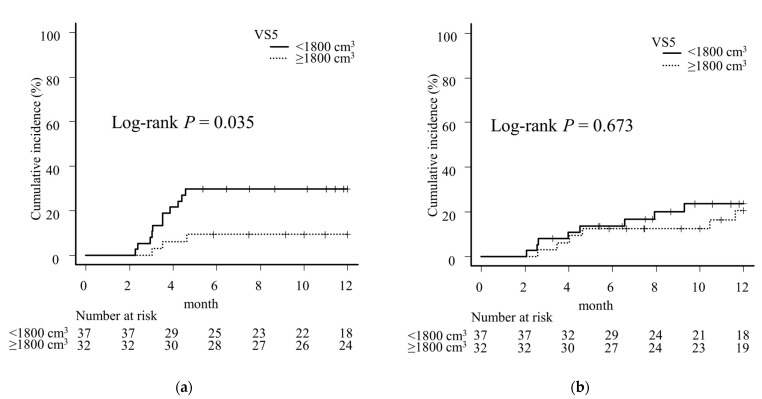
A comparison of the 12-month incidence of (**a**) pneumonitis within the irradiated field (in-field pneumonitis) grade ≥ 2 (≥G2) and (**b**) pneumonitis spreading beyond the irradiated field (out-of-field pneumonitis) ≥ G2 in Stage III non-small cell carcinoma patients treated with durvalumab consolidation after chemoradiotherapy using VS5 = 1800 cm^3^ as a cutoff value. Vertical lines on each line represent censored cases. In-field pneumonitis was significantly more common in VS5 < 1800 cm^3^ than ≥ 1800 cm^3^ (**a**), but no significant difference was seen in out-of-field pneumonitis (**b**).

In the univariate analysis, the 12-month incidence of pneumonitis ≥ G2 was significantly higher for a PF score ≥ 1 and MLD ≥ 14 Gy in Group N and VS5 < 1800 cm^3^ in Group D. Multivariate analysis showed that a PF score ≥ 1 (hazard ratio [HR] 2.89, 95% CI 1.21–6.91, *p* = 0.017) and MLD ≥ 14 Gy (HR 2.91, 95% CI 1.11–7.63, *p* = 0.030) were independent risk factors in Group N (Table 3), and VS5 < 1800 cm^3^ (HR 2.87, 95% CI 1.23–6.69, *p* = 0.015) was an independent risk factor in Group D (Table 4). On comparing the cumulative incidence of 12-month pneumonitis ≥ G2 in Group D using VS5 = 1800 cm^3^ as the cutoff value with Kaplan-Meier curves and log-rank tests, the incidence of in-field pneumonitis ≥ G2 was significantly higher in VS5 < 1800 cm^3^ cases (VS5 ≥ 1800 cm^3^, 9.4% vs. VS5 < 1800 cm^3^, 29.6%, *p* = 0.035). However, no significant difference was found among the cases of out-of-field pneumonitis ≥ G2 (VS5 ≥ 1800 cm^3^, 20.7% vs. VS5 < 1800 cm^3^, 23.6%, *p* = 0.673) (Figure 3).

## 4. Discussion

This study presents real-world data on durvalumab consolidation therapy after CCRT for stage III NSCLC in a single-center setting. Similar to the results of the PACIFIC study [3], PFS was significantly prolonged in patients treated with durvalumab consolidation therapy, although the difference was not statistically significant after PSM. Since durvalumab consolidation therapy has become the standard of care after CCRT for stage III lung cancer, several single- and multicenter studies have been conducted, many of which have confirmed its efficacy [6,7,21,22].

In the present study, durvalumab-treated patients had an increased incidence of pneumonitis ≥ G2 compared with CCRT-alone patients; however, this was mainly due to an increase in out-of-field pneumonitis, which was less closely related to the lung dose than in-field pneumonitis. This result supports the report by Xu et al. [8] comparing CCRT-alone with consolidation therapy with ICI after CCRT, and there was an association between MLD and treatment-related pulmonary adverse event grade ≥ 2 in the CCRT-alone group, whereas there was no association in patients treated with ICI consolidation. Although clinical data are scarce, pulmonary toxicity occurring outside the irradiated field is reportedly independent of the dosimetric parameters of the lungs in mouse experiments [23]. Radiation-induced organizing pneumonia has been reported to be one of the causes of out-of-field pneumonitis after thoracic radiotherapy, reportedly occurring in 1–2% of cases after postoperative radiotherapy for breast cancer [24,25,26,27] and in approximately 4% of cases after stereotactic body radiotherapy for lung cancer during the first year after treatment [28,29]. Another possible cause of out-of-field pneumonitis is the acute exacerbation of interstitial pneumonia. Our data also showed that PF score was an independent risk factor for pneumonitis ≥ G2 in Group N. The out-of-field pneumonitis in Group N was considered to be mainly due to these causes, and the increment in out-of-field pneumonitis in Group D was considered to be the effect of durvalumab, such as immune-related Adverse Events. Notably, in the present study, all cases of out-of-field pneumonitis in the no-durvalumab group were of grade ≥ 3, whereas more than half of the cases in the durvalumab group were of grade 2. Therefore, the prognosis of out-of-field pneumonitis caused by durvalumab is considered favorable.

Associations between the lung dose and the occurrence of pneumonitis after CCRT for NSCLC have been suggested. The report of Quantitative Analyses of Normal Tissue Effects in the Clinic recommended V20 ≤ 30–35% and MLD ≤ 20–23 Gy to maintain the incidence of pneumonitis below 20% for definitive treatment of NSCLC patients [11]. In the PACIFIC trial, MLD < 20 Gy and V20 < 35% were the lung dose limits [3]. Several associations between the lung dose and the occurrence of pneumonitis after durvalumab consolidation therapy have been reported. Yegya-Raman et al. [9] reported that lung V20 ≥ 28% was a risk factor for pneumonitis ≥ G2 in the ICI era. Other reports also showed that V20 was a risk factor for pneumonitis ≥ G2 in patients treated with durvalumab consolidation [30,31]. In a report by Masuo et al. [32], an analysis of 56 NSCLC patients who received durvalumab maintenance therapy after CCRT also showed that lung V20 was a risk factor for pneumonitis ≥ G2, in addition, the use of IMRT reduced the risk of pneumonitis ≥ G2. Mayahara et al. [17] reported that PF score ≥ 2 and V40 ≥ 10% were risk factors for pneumonitis ≥ G2. Tsukita et al. [33] reported a significant association between V5 and pneumonitis ≥ G2 in an analysis of patients treated entirely with IMRT. In contrast, as reported by Xu et al. [8], several reports have shown that pneumonitis in patients treated with ICI consolidation therapy, including durvalumab, is less related to the lung dose than in patients who did not receive ICI consolidation. Inoue et al. [34] reported that lung V20 was not a significant risk factor for the development of pneumonitis ≥ G2. In our study, a low VS5 level was a significant risk factor for in-field pneumonitis. To increase VS5, measures such as reducing V5 by adjusting the arc rotation angle or increasing TLV by deep-inspiration breath-hold irradiation can be considered. In the durvalumab era, we may again need to monitor the low-dose range, and when performing a DVH analysis, it is recommended that in-field pneumonitis and out-of-field pneumonitis be separately analyzed.

The most important limitation of this study was the difference in background factors between Groups D and N, especially the high proportion of VMAT and IFRT cases in Group D. A secondary analysis of RTOG0617 showed that the use of IMRT reduced the incidence of pneumonitis ≥ G3 [15], so it is possible that the incidence of pneumonitis ≥ G3 in the present study may have been underestimated due to the large number of VMAT cases in Group D. However, although there was no significant difference, Group D still tended to have better OS, PFS, and more pneumonitis ≥ G2 and out-of-field pneumonitis ≥ G2 than Group N after PSM, as was the case before PSM. These results suggest that differences in the distribution of IFRT or VMAT in the two groups may not have had that great an impact on outcomes. The relatively small sample size may have contributed to the lack of a significant difference in PFS after PSM. Another limitation is the short median observation period of approximately 20 months. Although this is a sufficient period to observe the occurrence of pneumonitis, it must be noted that it is rather short to compare the long-term treatment outcomes. We plan to conduct further studies with a longer observation period and an increased number of cases.

## 5. Conclusions

Durvalumab consolidation increased the incidence of out-of-field pneumonitis ≥ G2. However, there was no clear increase in severe pneumonitis and no fatal pneumonitis was observed. These results suggest that the prognostic benefit of durvalumab consolidation therapy outweighed the increased risk of pneumonitis. This study also showed that increasing VS5 levels in the lungs may reduce in-field pneumonitis ≥ G2.

## Figures and Tables

**Table 1:** Patient and treatment characteristics.

	Before PSM			After PSM		
	Durvalumab (Group D, n = 69)	No Durvalumab (Group N, n = 81)	*p*	Durvalumab (Group D, n = 30)	No Durvalumab (Group N, n = 30)	*p*
Sex			1.000			0.789
Male	48 (70)	57 (70)		20 (67)	18 (60)	
Female	21 (30)	24 (30)		10 (33)	12 (40)	
Age, median (range)	71 (44–84)	70 (48–87)	0.652	71 (57–77)	71 (48–85)	0.215
ECOG-PS			0.257			0.624
0	33 (48)	28 (35)		14 (47)	10 (33)	
1	32 (46)	46 (57)		14 (47)	17 (57)	
2	4 (6)	7 (9)		2 (7)	3 (10)	
Smoking history			0.817			0.706
Yes	60 (87)	69 (85)		27 (90)	25 (83)	
No	9 (13)	12 (15)		3 (10)	5 (17)	
PF score			0.058			0.117
0	60 (87)	62 (77)		25 (83)	23 (77)	
1	6 (9)	8 (10)		3 (10)	1 (3)	
2	2 (3)	11 (14)		1 (3)	6 (20)	
3	1 (1)	0 (0)		1 (3)	0 (0)	
Clinical stage (UICC 8th)			0.844			0.312
IIIA	37 (54)	40 (49)		19 (63)	13 (43)	
IIIB	24 (35)	32 (40)		8 (27)	11 (37)	
IIIC	8 (12)	9 (11)		3 (10)	6 (20)	
Histology			0.660			0.470
Squamous cell carcinoma	28 (41)	32 (40)		15 (50)	13 (43)	
Adenocarcinoma	35 (51)	40 (49)		14 (47)	13 (43)	
Others	6 (9)	9 (11)		1 (3)	4 (13)	
Treatment purpose			0.132			1.000
Definitive	53 (77)	69 (85)		26 (87)	27 (90)	
Salvage	16 (23)	12 (15)		4 (13)	3 (10)	
Driver gene mutation			0.416			0.671
Yes	5 (7) (EGFR 4, ALK 1)	10 (12) (EGFR 8, ALK 2)		2 (7) (EGFR 1, ALK 1)	4 (13) (EGFR 4)	
No	64 (93)	71 (88)		28 (93)	26 (87)	
PD-L1 TPS			<0.001			0.259
<1%	15 (22)	9 (11)		6 (20)	4 (13)	
1–50%	19 (28)	5 (6)		9 (30)	4 (13)	
50–100%	13 (19)	12 (15)		5 (17)	10 (33)	
Not available	22 (32)	55 (68)		10 (33)	12 (40)	
Radiation technique			<0.001			1.000
3D-CRT	24 (35)	69 (85)		19 (63)	18 (60)	
VMAT	45 (65)	12 (15)		11 (37)	12 (40)	
Radiation field			<0.001			1.000
ENI	13 (19)	63 (78)		13 (43)	12 (47)	
IFRT	56 (81)	18 (22)		17 (57)	18 (53)	
Prescription dose			0.505			0.612
<60 Gy	1 (1)	0 (0)		1 (3)	0 (0)	
=60 Gy	65 (94)	79 (98)		27 (90)	29 (97)	
>60 Gy	3 (4)	2 (2)		2 (7)	1 (3)	
PTV (cm^3^), median (IQR)	421 (316–583)	622 (409–903)	<0.001	566 (368–804)	601 (290–833)	0.929
TLV (cm^3^), median (IQR)	3348 (2680–4118)	3141 (2648–3884)	0.371	3163 (2652–4091)	2941 (2628–3746)	0.433
MLD (Gy), median (IQR)	12.3 (9.9–15.6)	13.8 (10.6–15.8)	0.155	12.8 (10.7–16.0)	13.9 (10.5–16.4)	0.706
V5 (%), median (IQR)	47.9 (39.7–56.6)	42.8 (35.7–51.0)	0.140	44.9 (41.5–55.8)	45.1 (35.5–56.5)	0.912
V20 (%), median (IQR)	22.0 (16.6–25.4)	24.4 (19.0–28.7)	0.066	22.8 (19.3–30.4)	25.6 (18.7–28.9)	0.690
VS5 (cm^3^), median (IQR)	1692 (1327–2310)	1708 (1365–2243)	0.908	1655 (1268–2152)	1591 (1337–1877)	0.717
Chemotherapy regimen			<0.001			0.208
CBDCA + PTX	30 (43)	12 (15)		11 (37)	6 (20)	
CDDP + TS-1	20 (29)	13 (16)		8 (27)	6 (20)	
CDDP + VNR	8 (12)	23 (28)		7 (23)	7 (23)	
CBDCA	9 (13)	18 (22)		4 (13)	6 (20)	
CBDCA + TS-1	0 (0)	6 (7)		0 (0)	1 (3)	
Others	2 (3)	9 (11)		0 (0)	4 (13)	

Data are presented as n (%), unless otherwise indicated. PSM, propensity score matching; ECOG-PS, Eastern Cooperative Oncology Group performance status; PF score, pulmonary fibrosis score; UICC, Union for International Cancer Control; EGFR, epidermal growth factor receptor; ALK, anaplastic lymphoma kinase; PD-L1, programmed cell death ligand 1; TPS, tumor proportion score; 3D-CRT, three-dimensional conformal radiotherapy; VMAT, volumetric modulated arc therapy; ENI, elective nodal irradiation; IFRT, involved field radiotherapy; PTV, planning target volume; IQR, interquartile range; TLV, total lung volume; MLD, mean lung dose; V5, percentage of lung volume irradiated with 5 Gy; V20, percentage of lung volume irradiated with 20 Gy; VS5, lung volume spared from 5 Gy.

**Table 2:** A comparison of lung doses in patients who developed pneumonitis grade ≥ 2 between out-of- and in-field pneumonitis.

	No Durvalumab (Group N)			Durvalumab (Group D)		
	Out-of-Field Pneumonitis (n = 4)	In-Field Pneumonitis (n = 17)	*p*	Out-of-Field Pneumonitis (n = 14)	In-Field Pneumonitis (n = 14)	*p*
MLD (Gy)	15.66 (12.95–17.33)	15.41 (12.36–17.45)	0.965	10.46 (9.18–14.02)	14.96 (12.30–17.48)	0.005
V5 (%)	63.69 (46.05–81.52)	49.11 (43.50–52.61)	0.275	43.57 (32.68–54.31)	51.07 (44.08–57.18)	0.074
V20 (%)	27.23 (23.91–29.34)	28.50 (21.51–33.29)	0.654	19.20 (14.56–24.56)	26.41 (20.68–31.17)	0.008
VS5 (cm^3^)	1241 (729–1649)	1517 (1444–1859)	0.395	1677 (1336–1999)	1516 (1320–1859)	0.486
TLV (cm^3^)	3120 (2290–3941)	3201 (2648–3700)	0.829	3025 (2488–3974)	3155 (2653–3694)	0.772

Data are presented as median (interquartile range), unless otherwise indicated. Out-of-field pneumonitis, pneumonitis spreading out of the irradiated field; In-field pneumonitis, pneumonitis within the irradiated field; MLD, mean lungs dose; Vx, x Gy to total lung volume; TLV, total lung volume; VS5, lung volume spared from 5 Gy.

**Table 3:** Results of univariate and multivariate analyses of pneumonitis grade ≥ 2 in Group N.

		Univariate		Multivariate	
Variables		HR (95% CI)	*p*	HR (95% CI)	*p*
Sex	Male	1.38 (0.51–3.77)	0.530		
	Female	[Reference]			
Age (years)	≥70	0.97 (0.41–2.29)	0.948		
	<70	[Reference]			
Smoking history	Yes	1.81 (0.42–7.79)	0.424		
	No	[Reference]			
PF score	0	[Reference]		[Reference]	
	1–3	3.53 (1.50–8.33)	0.004	2.89 (1.21–6.91)	0.017
Radiation technique	VMAT	1.15 (0.34–3.90)	0.826		
	3D-CRT	[Reference]			
Radiation field	IFRT	1.30 (0.48–3.55)	0.607		
	ENI	[Reference]			
PTV (cm^3^)	per 10 cm^3^	1.01 (1.00–1.02)	0.136		
Chemotherapy regimen	CDDP included	0.68 (0.29–1.61)	0.384		
	CBDCA included	[Reference]			
MLD (Gy)	≥14	3.48 (1.35–8.99)	0.010	2.91 (1.11–7.63)	0.030
	<14	[Reference]		[Reference]	
VS5 (cm^3^)	<1800	2.25 (0.91–5.59)	0.080		
	≥1800	[Reference]			

HR, hazard ratio; CI, confidence interval; PF score, pulmonary fibrosis score; VMAT, volumetric modulated arc therapy; 3D-CRT, three-dimensional conformal radiotherapy; IFRT, involved field radiotherapy; ENI, elective nodal irradiation, PTV, planning target volume; MLD, mean lung dose; VS5, lungs volume spared from 5 Gy.

**Table 4:** Results of univariate and multivariate analyses of pneumonitis grade ≥ 2 in Group D.

		Univariate		Multivariate	
Variables		HR (95% CI)	*p*	HR (95% CI)	*p*
Sex	Male	1.23 (0.54–2.79)	0.626		
	Female	[Reference]			
Age (years)	≥70	1.11 (0.52–2.38)	0.784		
	<70	[Reference]			
Smoking history	Yes	1.54 (0.47–5.11)	0.479		
	No	[Reference]			
PF score	0	[Reference]		[Reference]	
	1–3	1.25 (0.43–3.61)	0.677	1.70 (0.57–5.05)	0.340
Radiation technique	VMAT	1.16 (0.52–2.57)	0.716		
	3D-CRT	[Reference]			
Radiation field	IFRT	2.13 (0.64–7.06)	0.205		
	ENI	[Reference]			
PTV (cm^3^)	per 10 cm^3^	1.00 (0.99–1.02)	0.917		
Chemotherapy regimen	CDDP included	1.53 (0.73–3.21)	0.262		
	CBDCA included	[Reference]			
MLD (Gy)	≥14	0.97 (0.44–2.14)	0.939	0.67 (0.29–1.55)	0.351
	<14	[Reference]		[Reference]	
VS5 (cm^3^)	<1800	2.32 (1.05–5.13)	0.038	2.87 (1.23–6.69)	0.015
	≥1800	[Reference]		[Reference]	

HR, hazard ratio; CI, confidence interval; PF score, pulmonary fibrosis score; VMAT, volumetric modulated arc therapy; 3D-CRT, three-dimensional conformal radiotherapy; IFRT, involved field radiotherapy; ENI, elective nodal irradiation, PTV, planning target volume; MLD, mean lung dose; VS5, lung volume spared from 5 Gy.

## Data Availability

Data used in this study are available from the corresponding author upon reasonable request.

## Supplementary Materials

The following supporting information can be downloaded at: https://www.mdpi.com/article/10.3390/cancers16061162/s1, Figure S1: Kaplan-Meier curves of stage III non-small cell carcinoma patients treated by chemoradiotherapy with or without durvalumab consolidation therapy, cases in Group N for which treatment was initiated after July 2018 (since the start of durvalumab use) were excluded. Figure S2: Kaplan-Meier curves of stage III non-small cell carcinoma patients treated by chemoradiotherapy with or without durvalumab consolidation therapy after propensity score matching. Figure S3: Comparison of the 12-month incidence of pneumonitis after propensity score matching. Figure S4: Receiver operating characteristic (ROC) curves for the incidence of pneumonitis ≥ G2 in stage III NSCLC patients undergoing chemoradiotherapy.

## References

[1] Curran W.J., Paulus R., Langer C.J., Komaki R., Lee J.S., Hauser S., Movsas B., Wasserman T., Rosenthal S.A., Gore E. (2011). Sequential vs. concurrent chemoradiation for stage III non-small cell lung cancer: Randomized phase III trial RTOG 9410. J. Natl. Cancer Inst..

[2] Bradley J.D., Hu C., Komaki R.R., Masters G.A., Blumenschein G.R., Schild S.E., Bogart J.A., Forster K.M., Magliocco A.M., Kavadi V.S. (2019). Long-Term Results of NRG Oncology RTOG 0617: Standard- Versus High-Dose Chemoradiotherapy with or Without Cetuximab for Unresectable Stage III Non-Small-Cell Lung Cancer. J. Clin. Oncol..

[3] Antonia S.J., Villegas A., Daniel D., Vicente D., Murakami S., Hui R., Yokoi T., Chiappori A., Lee K.H., de Wit M. (2017). Durvalumab after chemoradiotherapy in stage III non-small-cell lung cancer. N. Engl. J. Med..

[4] Antonia S.J., Villegas A., Daniel D., Vicente D., Murakami S., Hui R., Kurata T., Chiappori A., Lee K.H., de Wit M. (2018). Overall survival with Durvalumab after chemoradiotherapy in stage III NSCLC. N. Engl. J. Med..

[5] Spigel D.R., Faivre-Finn C., Gray J.E., Vicente D., Planchard D., Paz-Ares L., Vansteenkiste J.F., Garassino M.C., Hui R., Quantin X. (2022). Five-year survival outcomes from the PACIFIC trial: Durvalumab after chemoradiotherapy in stage III non-small-cell lung cancer. J. Clin. Oncol..

[6] Girard N., Bar J., Garrido P., Garassino M.C., McDonald F., Mornex F., Filippi A.R., Smit H.J.M., Peters S., Field J.K. (2023). Treatment Characteristics and Real-World Progression-Free Survival in Patients with Unresectable Stage III NSCLC Who Received Durvalumab After Chemoradiotherapy: Findings From the PACIFIC-R Study. J. Thorac. Oncol..

[7] Jung H.A., Noh J.M., Sun J.M., Lee S.H., Ahn J.S., Ahn M.J., Pyo H., Ahn Y.C., Park K. (2020). Real world data of durvalumab consolidation after chemoradiotherapy in stage III non-small-cell lung cancer. Lung Cancer.

[8] Xu T., Wu L., Gandhi S., Jing W., Nguyen Q., Chen A., Chang J.Y., Nurieva R., Sheshadri A., Altan M. (2022). Treatment-related pulmonary adverse events induced by chemoradiation and durvalumab affect survival in locally advanced non-small cell lung cancer. Radiother. Oncol..

[9] Yegya-Raman N., Friedes C., Lee S.H., Iocolano M., Duan L., Wang X., Li B., Aggarwal C., Cohen R.B., Su W. (2023). Pneumonitis Rates Before and After Adoption of Immunotherapy Consolidation in Patients with Locally Advanced Non-Small Cell Lung Cancer Treated with Concurrent Chemoradiation. Int. J. Radiat. Oncol. Biol. Phys..

[10] Tsujino K., Hirota S., Endo M., Obayashi K., Kotani Y., Satouchi M., Kado T., Takada Y. (2003). Predictive value of dose-volume histogram parameters for predicting radiation pneumonitis after concurrent chemoradiation for lung cancer. Int. J. Radiat. Oncol. Biol. Phys..

[11] Marks L.B., Bentzen S.M., Deasy J.O., Kong F.S., Bradley J.D., Vogelius I.S., Naqa I.E., Hubbs J.L., Lebesque J.V., Timmerman R.D. (2010). Radiation dose-volume effects in the lung. Int. J. Radiat. Oncol. Biol. Phys..

[12] Tatsuno S., Doi H., Okada W., Inoue E., Nakamatsu K., Tanooka M., Tanaka M., Nishimura Y. (2022). Risk factors for radiation pneumonitis after rotating gantry intensity-modulated radiation therapy for lung cancer. Sci. Rep..

[13] Chen J., Hong J., Zou X., Lv W., Guo F., Hong H., Zhang W. (2015). Association between absolute volumes of lung spared from low-dose irradiation and radiation-induced lung injury after intensity-modulated radiotherapy in lung cancer: A retrospective analysis. J. Radiat. Res..

[14] Tsujino K., Hashimoto T., Shimada T., Yoden E., Fujii O., Ota Y., Satouchi M., Negoro S., Adachi S., Soejima T. (2014). Combined analysis of V20, VS5, pulmonary fibrosis score on baseline computed tomography, and patient age improves prediction of severe radiation pneumonitis after concurrent chemoradiotherapy for locally advanced non-small-cell lung cancer. J. Thorac. Oncol..

[15] Chun S.G., Hu C., Choy H., Komaki R.U., Timmerman R.D., Schild S.E., Bogart J.A., Dobelbower M.C., Bosch W., Galvin J.M. (2017). Impact of Intensity-Modulated Radiation Therapy Technique for Locally Advanced Non-Small-Cell Lung Cancer: A Secondary Analysis of the NRG Oncology RTOG 0617 Randomized Clinical Trial. J. Clin. Oncol..

[16] Kashihara T., Nakayama Y., Ito K., Kubo Y., Okuma K., Shima S., Nakamura S., Takahashi K., Inaba K., Murakami N. (2020). Usefulness of Simple Original Interstitial Lung Abnormality Scores for Predicting Radiation Pneumonitis Requiring Steroidal Treatment After Definitive Radiation Therapy for Patients with Locally Advanced Non-Small Cell Lung Cancer. Adv. Radiat. Oncol..

[17] Mayahara H., Uehara K., Harada A., Kitatani K., Yabuuchi T., Miyazaki S., Ishihara T., Kawaguchi H., Kubota H., Okada H. (2022). Predicting factors of symptomatic radiation pneumonitis induced by durvalumab following concurrent chemoradiotherapy in locally advanced non-small cell lung cancer. Radiat. Oncol..

[18] Shaverdian N., Thor M., Shepherd A.F., Offin M.D., Jackson A., Wu A.J., Gelblum D.Y., Yorke E.D., Simone C.B., Chaft J.E. (2020). Radiation pneumonitis in lung cancer patients treated with chemoradiation plus durvalumab. Cancer Med..

[19] Kazerooni E.A., Martinez F.J., Flint A., Jamadar D.A., Gross B.H., Spizarny D.L., Cascade P.N., Whyte R.I., Lynch J.P., Toews G. (1997). Thin-section CT obtained at 10-mm increments versus limited three-level thin-section CT for idiopathic pulmonary fibrosis: Correlation with pathologic scoring. AJR Am. J. Roentgenol..

[20] Kanda Y. (2013). Investigation of the freely available easy-to-use software ‘EZR’ for medical statistics. Bone Marrow Transplant..

[21] Ohri N., Halmos B., Bodner W.R., Cheng H., Garg M.K., Gucalp R., Guha C. (2021). Who benefits the most from adjuvant durvalumab after chemoradiotherapy for non-small cell lung cancer? An exploratory analysis. Pract. Radiat. Oncol..

[22] Kishi N., Matsuo Y., Shintani T., Ogura M., Mitsuyoshi T., Araki N., Fujii K., Okumura S., Nakamatsu K., Kishi T. (2023). Recurrence patterns and progression-free survival after chemoradiotherapy with or without consolidation durvalumab for stage III non-small cell lung cancer. J. Radiat. Res..

[23] Ghita M., Dunne V.L., McMahon S.J., Osman S.O., Small D.M., Weldon S., Taggart C.C., McGarry C.K., Hounsell A.R., Graves E.E. (2019). Preclinical evaluation of dose-volume effects and lung toxicity occurring in and out-of-field. Int. J. Radiat. Oncol. Biol. Phys..

[24] Katayama N., Sato S., Katsui K., Takemoto M., Tsuda T., Yoshida A., Morito T., Nakagawa T., Mizuta A., Waki T. (2009). Analysis of factors associated with radiation-induced bronchiolitis obliterans organizing pneumonia syndrome after breast-conserving therapy. Int. J. Radiat. Oncol. Biol. Phys..

[25] Murofushi-Nemoto K., Oguchi M., Gosho M., Kozuka T., Sakurai H. (2015). Radiation-induced bronchiolitis obliterans organizing pneumonia (BOOP) syndrome in breast cancer patients is associated with age. Radiat. Oncol..

[26] Ogo E., Komaki R., Abe T., Uchida M., Fujimoto K., Suzuki G., Tsuji C., Suefuji H., Etou H., Hattori C. (2010). The clinical characteristics and non-steroidal treatment for radiation-induced bronchiolitis obliterans organizing pneumonia syndrome after breast-conserving therapy. Radiother. Oncol..

[27] Sato H., Ebi J., Tamaki T., Yukawa A., Nakajima M., Ohtake T., Suzuki Y. (2018). Incidence of organizing pneumonia after whole-breast radiotherapy for breast cancer, and risk factor analysis. J. Radiat. Res..

[28] Murai T., Shibamoto Y., Nishiyama T., Baba F., Miyakawa A., Ayakawa S., Ogino H., Otsuka S., Iwata H. (2012). Organizing pneumonia after stereotactic ablative radiotherapy of the lung. Radiat. Oncol..

[29] Ochiai S., Nomoto Y., Yamashita Y., Murashima S., Hasegawa D., Kurobe Y., Toyomasu Y., Kawamura T., Takada A., Ii N. (2015). Radiation-induced organizing pneumonia after stereotactic body radiotherapy for lung tumor. J. Radiat. Res..

[30] Shintani T., Kishi N., Matsuo Y., Ogura M., Mitsuyoshi T., Araki N., Fujii K., Okumura S., Nakamatsu K., Kishi T. (2021). Incidence and Risk Factors of Symptomatic Radiation Pneumonitis in Non-Small-Cell Lung Cancer Patients Treated with Concurrent Chemoradiotherapy and Consolidation Durvalumab. Clin. Lung Cancer.

[31] Saito G., Oya Y., Taniguchi Y., Kawachi H., Daichi F., Matsumoto H., Iwasawa S., Suzuki H., Niitsu T., Miyauchi E. (2021). Real-world survey of pneumonitis and its impact on durvalumab consolidation therapy in patients with non-small cell lung cancer who received chemoradiotherapy after durvalumab approval (HOPE-005/CRIMSON). Lung Cancer.

[32] Masuo M., Shinohara E., Kitano M., Maruta R., Chonabayashi S., Endo S., Matumoto S., Nishiyama N., Machitori Y., Kobayashi M. (2023). A comparison of the incidence of ≥grade 2 radiation pneumonitis between intensity-modulated radiotherapy and three-dimensional conformal radiotherapy in patients with unresectable non-small cell lung cancer treated with durvalumab after concurrent chemoradiotherapy. Jpn. J. Clin. Oncol..

[33] Tsukita Y., Yamamoto T., Mayahara H., Hata A., Takeda Y., Nakayama H., Tanaka S., Uchida J., Usui K., Toyoda T. (2021). Intensity-modulated radiation therapy with concurrent chemotherapy followed by durvalumab for stage III non-small cell lung cancer: A multi-center retrospective study. Radiother. Oncol..

[34] Inoue H., Ono A., Kawabata T., Mamesaya N., Kawamura T., Kobayashi H., Omori S., Wakuda K., Kenmotsu H., Naito T. (2020). Clinical and radiation dose-volume factors related to pneumonitis after treatment with radiation and durvalumab in locally advanced non-small cell lung cancer. Investig. New Drugs.

