# Supplementary material for: Patterns and Incidence of Pneumonitis and Initial Treatment Outcomes with Durvalumab Consolidation Therapy after Radical Chemoradiotherapy for Stage III Non-Small Cell Lung Cancer
Source: Cancers (Basel). 2024 Mar 15;16(6):1162. doi: 10.3390/cancers16061162 (PMC10969599; doi:10.3390/cancers16061162)
Supplement: Supplementary file 1 [file cancers-16-01162-s001.zip › Supplementary_materials_final(clean).pdf]

Supplementary materials

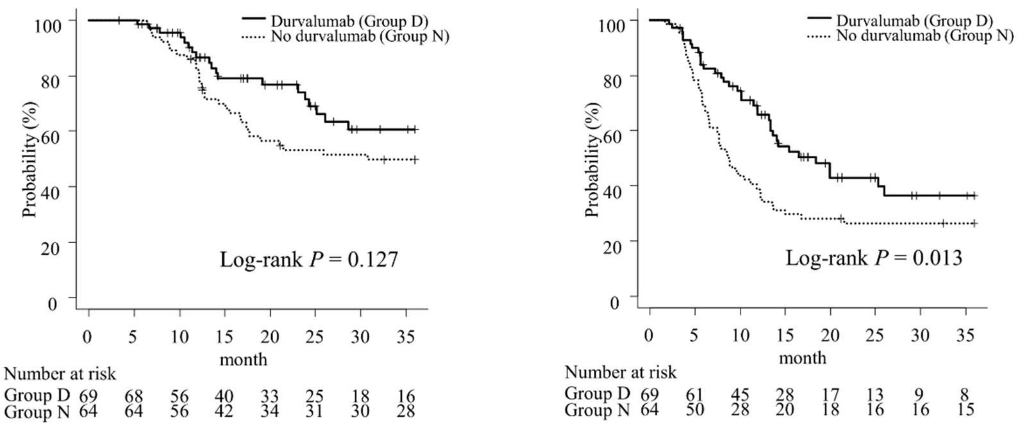

(**a**) (**b**)

Figure S1. Kaplan-Meier curves of stage III non-small cell carcinoma patients treated by chemoradiotherapy with or without durvalumab consolidation therapy, cases in Group N for which treatment was initiated after July 2018 (since the start of durvalumab use) were excluded. Vertical lines on each line represent censored cases. (a) Overall survival (OS). (b) Progression-free survival (PFS). Although there was no significant difference in OS (a), the PFS was significantly better in the durvalumab group than in the no-durvalumab group (b).

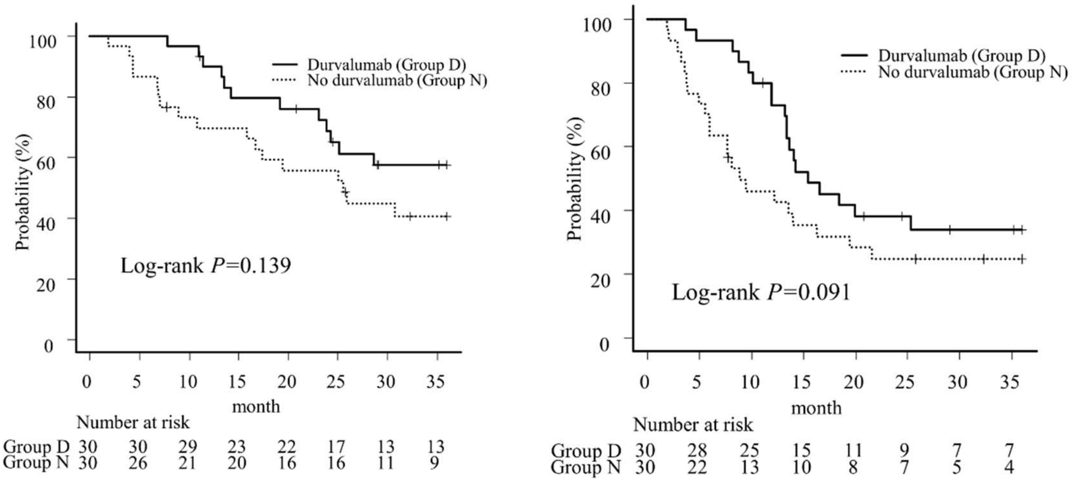

(**a**) (**b**)

Figure S2. Kaplan-Meier curves of stage III non-small cell carcinoma patients treated by chemoradiotherapy with or without durvalumab consolidation therapy after propensity score matching. Vertical lines on each line represent censored cases. (a) Overall survival (OS). (b) Progression-free survival (PFS). Both OS and PFS tended to be better in Group D than Group N, but the differences were not significant.

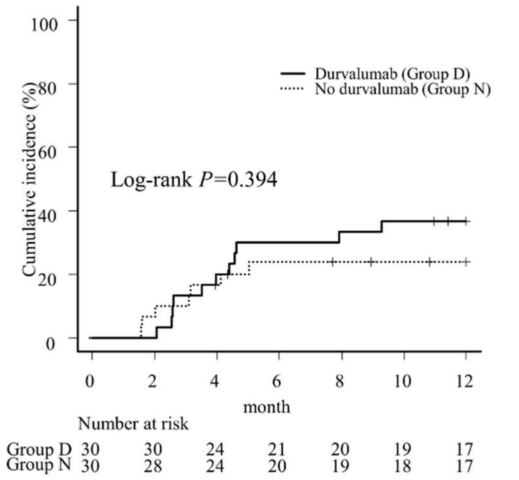

(**a**)

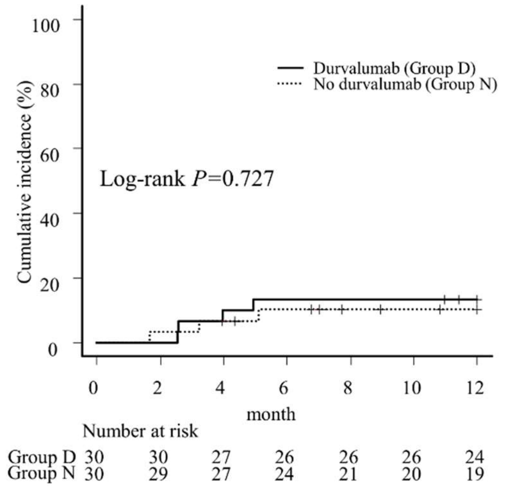

(**b**)

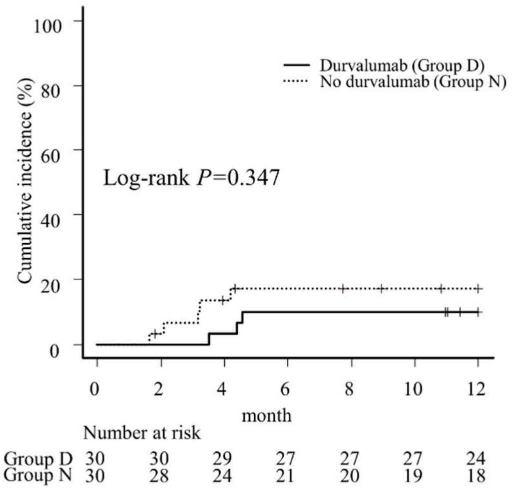

(c)

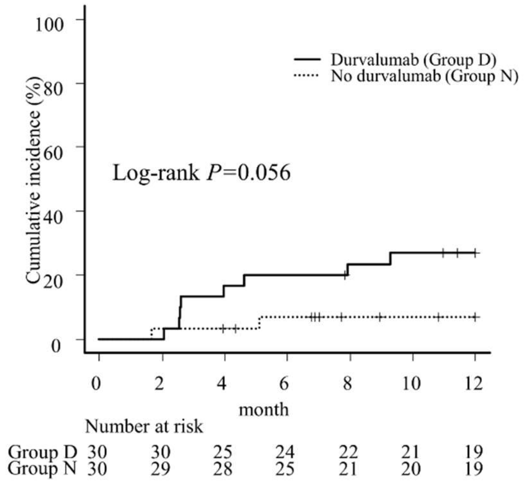

(d)

Figure S3. Comparison of the 12-month incidence of pneumonitis after propensity score matching. (a) pneumonitis grade ≥2 (≥G2), (b) pneumonitis grade ≥3 (≥G3), (c) pneumonitis within the irradiated field (in-field pneumonitis) ≥G2, and (d) pneumonitis spreading beyond the irradiation field (out-of-field pneumonitis) ≥G2 in stage III non-small cell carcinoma patients treated with or without consolidation durvalumab after chemoradiotherapy. Vertical lines on each line represent censored cases. There was a trend toward more pneumonitis ≥G2 in the durvalumab group than in the no-durvalumab group, the difference was not significant (a). There was no marked difference between the groups in pneumonitis ≥G3 (b). The incidence of in-field pneumonitis ≥G2 did not differ between the two groups (c). Out-of-field pneumonitis tended to be more common in the durvalumab group than in the no-durvalumab group; however, the difference was not significant (d).

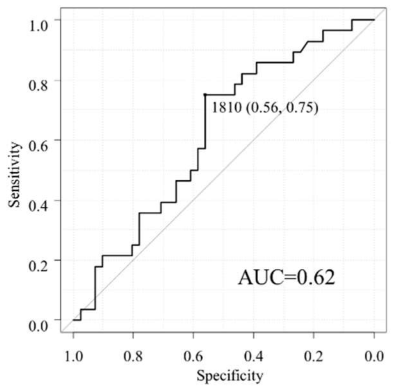

(a)

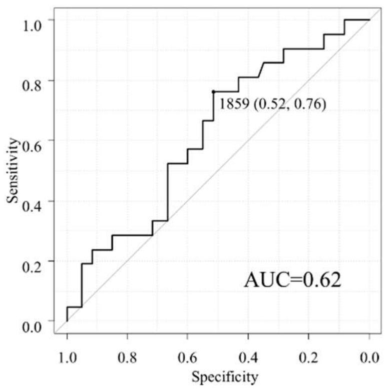

(b)

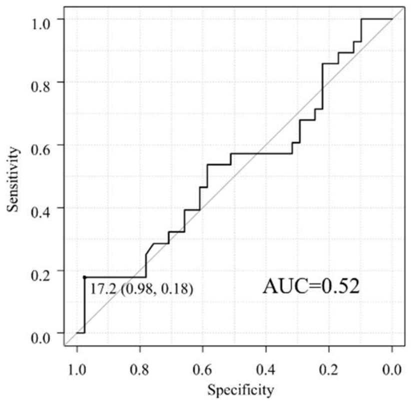

(c)

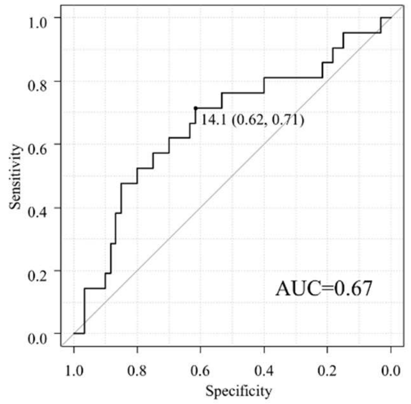

(d)

Figure S4. Receiver operating characteristic (ROC) curves for the incidence of pneumonitis ≥G2 in stage III NSCLC patients undergoing chemoradiotherapy. (a) lung VS5 for Group D, (b) lung VS5 for Group N, (c) MLD for Group D, (d) MLD for Group N. The AUC was 0.62 and Youden's index was 1810 cm^3 for Group D (a). The AUC was 0.62 and Youden's index was 1859 cm^3 for Group N (b). The AUC was 0.52 and Youden's index was 17.2 Gy for Group D (c). The AUC was 0.67 and Youden's index was 14.1 Gy for Group N (d). VS5, lung volume spared from 5Gy; MLD, mean lung dose; AUC, area under the curve.
